# Nuclear factor kappa B is required for the production of infectious human herpesvirus 8 virions

**DOI:** 10.3389/fmicb.2014.00129

**Published:** 2014-04-04

**Authors:** Negin N. Blattman, Michael Lagunoff, Joseph N. Blattman, Lawrence Corey

**Affiliations:** ^1^Molecular and Cellular Biology Program, University of Washington School of MedicineSeattle, WA, USA; ^2^Infectious Diseases and Vaccinology, Arizona State UniversityTempe, AZ, USA; ^3^Departments of Microbiology, University of Washington School of MedicineSeattle, WA, USA; ^4^Vaccine and Infectious Disease Division, Fred Hutchinson Cancer Research CenterSeattle, WA, USA; ^5^Departments of Medicine, University of Washington School of MedicineSeattle, WA, USA; ^6^Departments of Laboratory Medicine, University of Washington School of MedicineSeattle, WA, USA

**Keywords:** HHV8, KSHV, NFκB, HF, MVEC, BCBL-1

## Abstract

Human herpesvirus 8 (HHV8) infection leads to potent activation of nuclear factor kappa B (NFκB) in primary and transformed cells. We used recombinant HHV8 (rKSHV.219) expressing green fluorescent protein under the constitutive cellular promoter elongation factor 2α and red fluorescent protein under an early HHV8 lytic gene promoter T1.1 to monitor replication during infection of human foreskin fibroblasts (HF), noting changes in NFκB activity. In primary HF, NFκB levels do not affect the ability of HHV8 to establish infection or maintain latency. Furthermore, there was no effect on the percent of cells undergoing reactivation from latency, and there were similar numbers of released and cell-associated HHV8 viral particles following reactivation in the presence of inhibitors. Reactivation of HHV8 in latently infected HF in the presence of NFκB inhibitors resulted in production of viral particles that did not efficiently establish infection, due to deficiencies in binding and/or entry into normally permissive cells. Exogenous expression of glycoprotein M, an envelope protein involved in viral binding and entry, was able to partially overcome the deficiency induced by NFκB inhibitors. Our data indicate that in primary cells, NFκB is not required for infection, establishment of latency, or entry into the lytic cycle, but is required for the expression of virion associated genes involved in the initial steps of virion infectivity. These studies suggest that strategies to inhibit NFκB may prevent HHV8 spread and should be considered as a potential therapeutic target for preventing HHV8 associated diseases.

## INTRODUCTION

Human herpesvirus 8 (HHV8), also called Kaposi’s sarcoma associated herpesvirus (KSHV), is a deoxyribonucleic acid (DNA) tumor virus that is epidemiologically and molecularly linked to multiple proliferative disorders including: endothelial cell based tumor Kaposi’s Sarcoma (Soulier et al., 1995); B cell tumors; pleural effusion lymphoma (PEL), also known as body cavity based lymphoma (BCBL); and the plasmablastic variant of Multicentric Castleman’s Disease (Inglesby et al., 1999). Persistent HHV8 infection is characterized by both latency and lytic replication, and diseases associated with HHV8 infection appear to be the result of both long-term latent infection and viral reactivation. Latent infection is associated with the absence of virus production and the expression of a limited number of viral genes, many of which provide a survival advantage (Dittmer et al., 1998; Ensoli et al., 2001; Fakhari and Dittmer, 2002). Lytic replication is characterized by the timely expression of immediate early, early, and late genes resulting in the release of mature virions. Lytic replication is thought to aid in tumorigenesis by increasing the pool of infected cells and lytic genes may also have paracrine properties that might provide neighboring cells with proliferation and/or survival advantage (Dupin et al., 1999; Ensoli et al., 2001; Boulanger et al., 2004).

Nuclear factor kappa B (NFκB) is an important transcription factor involved in the lifecycle of many herpesviruses, including HHV8 (Keller et al., 2000; Hummel et al., 2001; Hummel and Abecassis, 2002; Guasparri et al., 2004; Stewart et al., 2004; Sadagopan et al., 2007; Grossmann and Ganem, 2008). NFκB is involved in the cellular processes like inflammation, cell proliferation, protection from apoptosis, and viral gene expression. Deregulation of NFκB has been linked to lymphomas caused by Epstein-Barr Virus (EBV) infection as well as viral reactivation in Cytomegalovirus (CMV) infected cells (Hummel et al., 2001; Hummel and Abecassis, 2002; Stewart et al., 2004). HHV8 has multiple NFκB binding sites in its genome regulating both latent and lytic genes, and NFκB activity is up regulated during infection (Keller et al., 2000; Lagunoff et al., 2001; Guasparri et al., 2004; Sadagopan et al., 2007; Grossmann and Ganem, 2008). NFκB has been implicated as a survival factor for HHV8 latently infected cells and thought to be involved in HHV8 lytic replication (Keller et al., 2000; Guasparri et al., 2004; Sgarbanti et al., 2004; Grossmann and Ganem, 2008).

A number of cell culture systems in which to study HHV8 replication and the role of NFκB have been used. BCBL-1 cells, a line created from a PEL, have been used to study HHV8 extensively; however, BCBL-1 cells predominantly maintain HHV8 in the latent state, with a low percentage of lytically replicating cells at all times. As a population, the cells exhibit high levels of constitutive NFκB activity. Reduction of NFκB levels in PEL cells by specific inhibitors results in apoptosis and increased sensitivity to apoptotic stimuli (Guasparri et al., 2004). However, some studies have suggested that increased NFκB levels are required for lytic gene expression (Sgarbanti et al., 2004; Grossmann and Ganem, 2008). Additional work has shown NFκB has inhibitory effects on lytic viral gene promoters, suggesting that NFκB suppresses lytic viral gene expression (Brown et al., 2003). Grossmann and Ganem (2008) confirmed the dependence of PEL cells on NFκB for maintenance of latency, and found that lytic gene expression was enhanced during blockade of NFκB. Consistent with these results, blockade of NFκB in PEL cells correlated with the increased expression of the lytic genes ORF K8.1 and ORF 59 (Brown et al., 2003). Additionally, Grossmann and Ganem (2008) demonstrated that this does not seem to be universal, as human foreskin fibroblasts (HF) and microvascular endothelial cells (MVEC) do not exhibit this phenotype after NFκB inhibition, for the first time demonstrating that NFκB dependence may be cell type specific. Furthermore, since B-cell transformation is often dependent upon deregulation of NFκB, NFκB induced events leading to viral reactivation in transformed B cells may not adequately reflect cellular events that occur during viral reactivation in non-transformed primary cells. Sadagopan et al. (2007) found that NFκB levels increased within minutes of infection with HHV8 and remained elevated, directly affecting latent and lytic gene expression levels. They did not however, evaluate the role of NFκB during viral reactivation and virion production. Given these conflicting results about the role of NFκB in transformed cells it is important to examine the role of NFκB in viral reactivation and virus production during HHV8 infection of primary human cells that survive independent of NFκB signaling.

To assess the role of NFκB in HHV8 infection, particularly during viral reactivation, we utilized an HHV8 infection model previously developed in primary HF as well as an additional model using MVEC (Lagunoff et al., 2002; Vieira and O’Hearn, 2004). We found that NFκB levels increase shortly after infection, remain elevated during latency, and increase dramatically during reactivation. Interestingly, while in the presence or absence of NFκB inhibitors an equivalent percentage of cells underwent viral reactivation and similar numbers of virus particles were produced; however, there was a stark decrease in the production of infectious virions. This effect could be partially rescued by expression of the KSHV lytic gene gM.

## MATERIALS AND METHODS

### CELL CULTURE AND NFκB INHIBITORS

Human foreskin fibroblasts (HF) and 293 cells were grown in Dulbeco’s Modified Eagles Media supplemented with 10% heat inactivated FBS and 1% pen/strep. MVEC were grown in endothelial basal medium-2 with bullet kit additives (Lonza). Stable rKSHV.219 infection was established by infection of HF cells with a multiplicity of infection (MOI) of 10. rKSHV.219 contains a puromycin resistance cassette and after 72 h the infected cells were selected with puromycin. HF cells infected with rKSHV.219 were maintained under the same selection (Lagunoff et al., 2002; Vieira and O’Hearn, 2004). In order to inhibit NFκB activity two methods were chosen. First, where indicated, cells were treated with the irreversible small molecule inhibitor of NFκB, Bay11-7082, at 5 μM and 2.5 μM to HF and MVEC cells, respectively. Alternatively, we also utilized a dominant negative form of the inhibitor of NFκB(IκBα) containing two point mutations (32/36S/A) expressed via a DNA vector (pCMV-IκBα-DN, courtesy of D. Ballard, Vanderbilt University). One μg of a Cyan fluorescent protein expression vector was used as a transfection efficiency control. Bay11-7082 treatments were as follows: cells were treated with 5 or 2.5 uM Bay11-7082 or a DMSO control for 24 h prior to the start of the experiment and for the duration of the experiment; for the “Post” samples, cells were treated with 5 or 2.5 uM Bay11-7082 at time of induction of lytic replication and for the duration of the experiment.

### VIRAL INFECTION, INDUCTION AND QUANTITATION

Recombinant HHV8 construction and infection of HF, viral replication induction and viral titer quantitation were performed as previously described (Vieira and O’Hearn, 2004). Infection of MVEC was performed in 12 well plates at an MOI of 10 (Lagunoff et al., 2002). Two h post infection, viral reactivation was induced using a recombinant adenovirus expressing the gene for the viral transactivator ORF50 [a gift from D. Ganem, UC, San Francisco (Glaunsinger)] in the presence of sodium butyrate and after 24 h the cells were washed with PBS and media replaced. Viral titer was determined with cell debris free supernatant incubated with 293 cells as previously described (Vieira and O’Hearn, 2004; Chen and Lagunoff, 2007). In all groups, mock inductions with sodium butyrate were performed as negative controls. To measure cell-associated virus, cells were harvested and the cell fraction was sonicated on ice for 10 s at 3-min intervals (repeated three times). Cell debris was pelleted and supernatant used to infect 293 cells. Original supernatant was used to quantitate viral titer.

### NFκB LUCIFERASE REPORTER ASSAY

Human foreskin fibroblasts were electroporated with the following luciferase reporter vectors then plated at 2 x 10^5^ cells/well in 12-well plates: pF-Luc (10 μg), a control vector containing two mutated NFκB consensus sites unable to bind NFκB; and pBXII-Luc (10 μg), which contains two tandem repeats of the NFκB consensus sites to reflect NFκB activity. A β-galactosidase (βgal) expression vector driven by a CMV promoter (1 μg) was used to measure transfection efficiency. To control for NFκB specific reporter activity, we utilized a dominant negative version of the NFκB inhibitor IκBα-DN (5 μg), which contains serine to alanine substitutions at amino acid positions 32 and 36, preventing phosphorylation and subsequent release of NFκB for translocation into the nucleus. Cells were harvested according to Promega Luciferase Assay Reagent protocol (E4030) 24 h post transfection and assayed for luminescence (TD Luminometer). Each transfection was performed in triplicate and read three times.

### IMMUNOHISTOCHEMISTRY AND FLOW CYTOMETRY

Cells were harvested at 72 h post induction of viral replication. All staining was performed on ice in PBS, 1% BSA, and 0.1% sodium azide. Cells were washed twice with PBS then stained with the K8.1 primary antibody at 1:100 in 5% FBS for 1 h. Cells were washed three times with PBS and stained with the secondary antibody anti-mouse Alexa 680 (Molecular Probes) at 1:1000 for 30 min then washed. Microscopy was performed with the Nikon Eclipse TE300 inverted fluorescent microscope equipped with filter sets TE300 FITC, TE300 Texas Red HYQ, and TE300 633. Images were acquired with a Photometrics CoolSnap cf digital camera and MetaVue imaging software at 20X amplification. Cells were analyzed by flow cytometry on an LSRII (Beckman-Coulter).

### ELECTROMOBILITY SHIFT ASSAYS

A quantity of 5 × 10^6^ HF and HF219 cells were washed in cold PBS before use of the NFκB specific Promega Gel Shift Assay System (E3050, E3300) utilizing the Promega NFκB specific probe (5′-AGT TGA GGG GAC TTT CCC AGG C-3′) as directed. For super-shift experiments, nuclear extracts were incubated with human p65 (Santa Cruz Biotechnology) for 30 min on ice prior to addition of radiolabeled oligonucleotide and gel electrophoresis. Samples were run on 7% acrylamide gels and assessed by exposure to blue x-ray film (Phenix Research Products).

### QUANTITATION OF VIRIONS

Supernatant harvested from uninduced and induced cells were filtered through 0.45 μm filters then centrifuged in rotor SS35 at 23 K RPM for 2 h at 4° C. The pellet was DNase treated for 1 h. As an internal control for non-encapsulated viral DNA, naked HSV gB (herpes simplexvirus glycoprotein B) DNA was added in known quantity to samples prior to DNAse treatment and subsequently analyzed by qPCR for HHV8 genes ORF73 and HSV gB (Molecular Virology Laboratories). Viral DNA copy number in 293 cells was measured by inducing HF219 cells, harvesting the supernatant and infecting 293 cells as previously described above. Four h post-infection cells were washed with PBS then DNAse treated as above. DNA from the final wash mixed with 293 cells was used for quantitative HHV8 PCR (ORF73), normalizing to beta globin (Molecular Virology Laboratories, UW).

### VIRAL GENE ARRAYS

Content of the viral arrays has been previously described (Chen and Lagunoff, 2007). The nitrocellulose membrane arrays contained 89 HHV8 genes and GAPDH and actin as cellular RNA controls. Messenger RNA was extracted using Qiagen RNeasy and Oligotex kits from control and experimental groups. cDNA was made using superscript II (Invitrogen) with random primers and P^32^ labeled dCTP. Labeled cDNA was then used to probe viral arrays overnight. The blots were then exposed to a phosphoimager screen and hybridization quantitated using a Typhoon scanner.

### REAL TIME PCR

Applied Biosystems program Primer Express 1.5 was used to design primers based on the sequences for all seven genes identified by viral arrays as well as T1.1 and K8.1 control genes. Supplementary Table 1 lists the primers used for this study. RNA was extracted using the Qiagen RNeasy kit. Briefly, 20 ug of RNA was treated with amplification grade DNase (Invitrogen) for 30 min followed by random primer reverse transcriptase PCR (100 uL reaction) and 2 uL was used for rRNA and 5 uL for gene specific amplification. Primers were first tested on uninfected HF and uninduced HF219 cells to ensure specificity. Ribosomal RNA was quantitated using a ribosomal RNA reagents kit with VIC probes and SYBER Green master mix (Applied Biosciences). Reactions were performed in an ABI Prism 7700 sequence detector. PCR results were normalized to rRNA levels.

### gM COMPLEMENTATION

Deoxyribonucleic acid encoding glycoprotein M was amplified using the primers 5′-TGAAAACAGCAGCATTTCCAA-3′ and 5′-TACTGACTCGGTGAAACC-3′. The PCR product was blunt ligated into the pIRES2 vector (Lonza) and constructs were sequenced to ensure correct coding sequence and orientation. Five micrograms of vector alone or gM construct were electroporated into cells and plated at 2 x 10^5^ cells per well and 24 h later cells were harvested and RNA extracted as described above. gM complementation was evaluated by inducing lytic replication and virus titer 24 h after transfecting cells.

## RESULTS

### INFECTION WITH rKSHV.219 INDUCES NFκB SIGNALING

**FIGURE 1:**
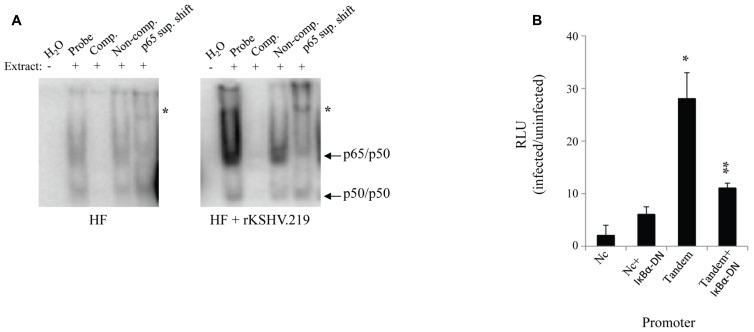
**rKSHV.219 leads to constitutive NFκB activation in HF**. **(A)** HF cells were either mock infected or infected with rKSHV.219 at a MOI of 10. At 72 h post-infection 2 x 10^5^ cells were harvested and 2 uL of nuclear extracts were characterized by EMSA using an NFκB–specific radiolabeled probe. Lane 1, H_2_O; 2, Nuclear extract (NE); 3, cold NFκB 50-fold molar excess probe (Comp.); 4, Non-competitive probe (Non-comp.); and 5, super shift with an anti-p65 antibody (p65 sup. Shift; *). Arrows denote protein subunits p50 and p65. This is a representative of three separate experiments. **(B)** HF cells were transfected with NFκB-driven luciferase plasmids: pF-Luc, mutated/nonconsensus NFκB binding sites (Dupin et al., 1999); and pBXII-Luc, two tandem consensus NFκB binding sites (Tandem). When indicated, cells were also cotransfected with IκBα-DN (Fenner et al., 1998). Cells were infected with rKSHV.219 and luciferase values were enumerated. All values reflect increases over uninfected controls, which were set to 1. Error bars represent standard deviations of the mean, calculated from one representative experiment performed in triplicate. *p < 0.0001 infected vs. uninfected fibroblasts, and **p < .0001 infected vs. uninfected expressing IκBα-DN.

We assessed the impact of HHV8 infection on active NFκB levels in primary HF. Cells were infected with rKSHV.219 at a MOI of 10 green fluorescent protein (GFP) forming units; this MOI is known to result in adequate levels of infection (30-50%) with little cell toxicity, a lower MOI results in inefficient infection of HF cells. GFP forming units were calculated by rKSHV.219 virus titer on 293 cells as previously described as a measure of MOI (Vieira and O’Hearn, 2004). Cell lysates were harvested 72 h post- infection and NFκB activity, assessed as binding to promoter DNA, was assayed by electromobility shift assay. NFκB consensus sites (5′-GGGRNNYYCC-3′) are present at several locations within the HHV8 genome, particularly upstream of lytic genes. We found that upon infection with rKSHV.219, HF cells had a relative increase in nuclear translocation of NFκB as demonstrated by electromobility shift assay (EMSA; **Figure 1A**). To determine specificity of the assay, antibody directed against the *p*65 subunit of NFκB was added. In order to enumerate NFκB activity we transfected HF cells accordingly with plasmids driven by either mutated, non-consensus NFκB sites (Dupin et al., 1999) or two tandem NFκB sites (Tandem) driving luciferase. A β-galactosidase transfection control was used to normalize for transfection efficiency. A subset of cells were cotransfected with a dominant negative IκBα (IκBα-DN), a mutated form of the NFκB inhibitor that contains two point mutations (S32A and S36A), which remove phosphorylation sites required for proteasome degradation, thereby resulting in constitutive NFκB inhibition. Expression of IκBα-DN led to decreased NFκB signaling by sequestering NFκB in the cytoplasm. Cells infected with rKSHV.219 had nearly a 28-fold increase in NFκB driven luciferase activity as compared to uninfected controls (**Figure 1B**). This increase in luciferase gene expression was partially inhibited by IκBα-DN.

### INHIBITION OF NFκB DOES NOT AFFECT LYTIC GENE EXPRESSION AND VIRAL REACTIVATION

**FIGURE 2:**
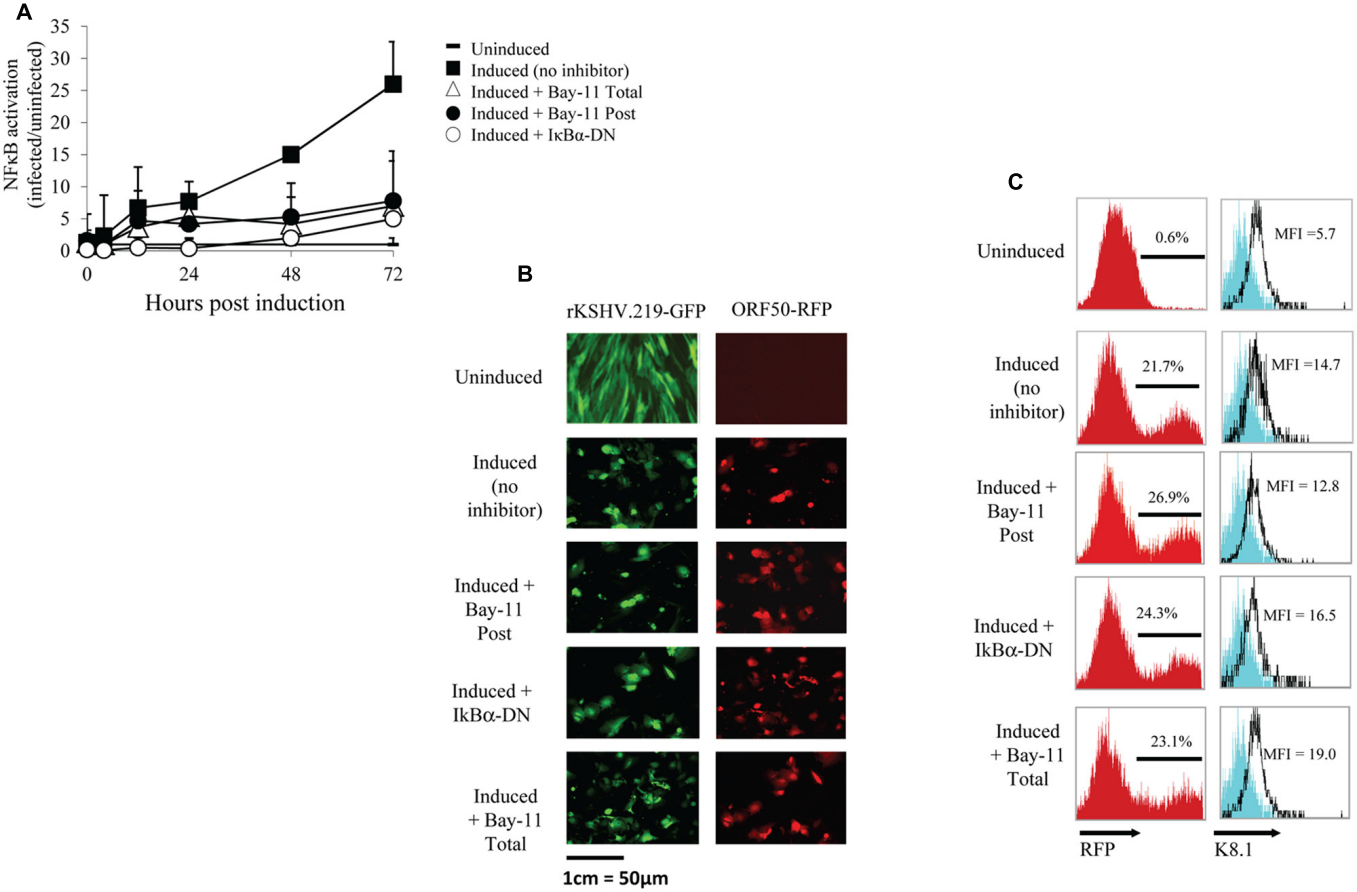
**NFκB inhibition does not affect viral reactivation**. **(A–C)** HF cells either mock infected or infected with rKSHV.219, transfected with pBXII-Luc and then induced to undergo lytic replication, except for the Uninduced sample. When indicated, cells were either mock treated (No inhibitor), cotransfected with IκBα-DN or treated with Bay11-7082 prior to induction (Total) or at time of induction (Post). **(A)** Cell lysates were harvested; luciferase values reflect NFκB activation as the fold-increase of infected/uninfected samples (set to 1); mean ± SD from triplicate transfections in one experiment, representative of three independent experiments. Student’s *t*-test was used for statistical analysis. **(B)** Cells were harvested and GFP expression (indicating infection) and RFP expression (RFP driven by ORF50 promoter, indicating viral reactivation) was visualized via fluorescence microscopy at 20X magnification. **(C)** Histograms showing the percentage of RFP positive cells and K8.1 protein expression as measured by flow cytometry; histogram values represent mean fluorescence intensity (MFI) of 5 x 10^6^ cells. The cells shown on the histogram are gated for GFP positive cells, which represents >99% of cells analyzed as all cells are maintained under puromycin selection. Data are representative of three independent experiments.

To further investigate NFκB activity during the viral life cycle we measured NFκB-dependent gene expression during viral reactivation. We either mock infected or infected HF cells with rKSHV.219 at an MOI of 10. rKSHV.219 contains a puromycin resistance cassette and infected cells were selected for puromycin resistance until cells were confluent, approximately 7 days later (Vieira and O’Hearn, 2004). Infected and mock-infected HF cells were electroporated with luciferase constructs as described above. Both cell populations were transfected with IκBα-DN-containing or empty vectors and were then induced to undergo productive lytic replication. It has previously been shown that ectopic expression of HHV8 ORF50 by a recombinant baculovirus (BacK50) in HF cells induces the virus from a latent to a lytic, replicating state, and that sodium butyrate significantly enhances ORF50-dependent virus production (Vieira and O’Hearn, 2004). Since transfection efficiency in primary HF cells is approximately 30%, we also utilized a non-reversible small molecule inhibitor of NFκB, Bay11-7082, and compared its effect on NFκB activity with that of IκBα-DN. We did evaluate the cell toxicity of Bay11-7082 by performing a dose response assay and found optimal inhibition of NFκB and minimal cell toxicity at 5 μM (data not shown). Where indicated, cells were treated with 5 μM Bay11-7082 or DMSO either 24 h prior to induction of lytic replication (Total) or at the time of induction (Post). We induced lytic replication of rKSHV.219 in HF cells (and mock infected cells) by infecting with BacK50 at an MOI of 40 as previously described (Vieira and O’Hearn, 2004), harvested cell lysates 4, 12, 24, 48, and 72 h post induction of lytic replication, and measured luciferase activity. After normalizing for transfection efficiency, we observed NFκB-driven luciferase expression at 4 h post induction, and by 72 h had increased to 25-fold higher than that of uninfected cells (**Figure 2A**). Treatment of cells with Bay11-7082 or transfection with IκBα-DN significantly inhibited NFκB driven luciferase activity, decreasing it by 5-fold.

We next sought to determine if inhibition of NFκB by Bay11-7082 or IκBα-DN resulted in spontaneous reactivation or inhibition of viral reactivation. HF cells infected with rKSHV.219 were either treated with Bay11-7082 or electroporated with IκBα-DN, and 24 h later induced to undergo lytic replication. After 48 h, cells were harvested and analyzed for GFP and RFP expression by microscopy, as well as RFP by flow cytometry. All cells infected with rKSHV.219 fluoresced green and uninduced cells did not undergo reactivation, as evidenced by lack of RFP expression (**Figure 2B**). Amongst the groups induced to undergo lytic replication, there was no difference in the number of RFP positive cells between the 3 treatment groups, suggesting that inhibition of NFκB does not alter the ability of rKSHV.219 to enter the lytic cycle (**Figure 2C**). To determine if late gene expression, in addition to early gene T1.1 expression, was affected by NFκB inhibition, we assayed for expression of the HHV8 late gene ORF K8.1 (**Figure 2C**). There was no significant difference in K8.1 protein expression in control, Bay11-7082 treated or IκBα-DN transfected cells. Interestingly, neither the T1.1 nor ORFK8.1 promoters contain an NFκB consensus site (Russo et al., 1996). Treatment of rKSHV.219 infected HF cells with NFκB inhibitors did not lead to spontaneous reactivation (**Figure 2B**, Vieira and O’Hearn, 2004).

### NFκB IS REQUIRED TO PRODUCE INFECTIOUS VIRIONS

**FIGURE 3:**
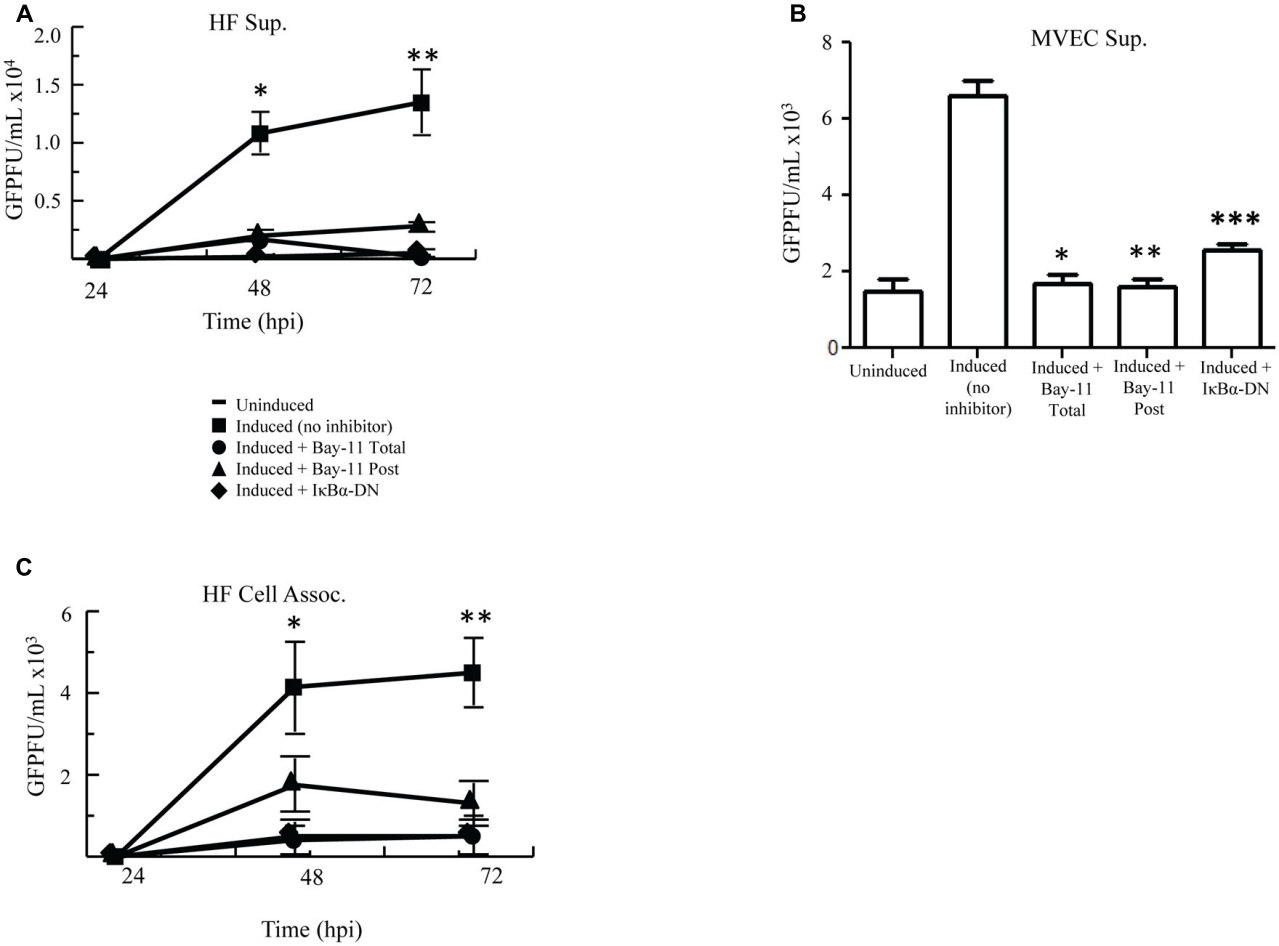
**Cell induction in the presence of NFκB inhibition decreases viral titers**. **(A,B)** rKSHV.219-infected HF **(A)** or MVEC **(B)** cells were either mock induced (Uninduced) or induced to undergo lytic replication with BacK50 **(A)** or Ad50 **(B)** and then treated with Bay11-7082 prior to induction (Total), at the time of induction (Post), or transfected with IκBα-DN prior to induction. Supernatants were harvested at 24, 48, and 72 h post induction **(A)** and GFP forming units on 293 cells were measured. Using paired *T* test there was statistical significance between control induction and all treatment groups at both 48 h *p < 0.032, 0.0371, and 0.033; and 72 h **p < 0.03, 0.033, 0.037. **(B)** MVEC titers at 72 h post induction assessed on 293 cells. P values calculated as compared with control inductions done with Ad50. *p < 0.0071, **p < 0.0035, ***p < 0.007. **(C)** Viral titers, measured by GFP forming units, from HF cell lysates described in (A). Using paired *T* test there was statistical significance between control induction and all treatment groups at 48 h *p < 0.045, 0.022, 0.0088 and < 0.03; and 72 h **p < 0.007, 0.002, 0.001. Samples were assayed in triplicate and are representative of three experiments.

Because NFκB inhibition in PEL cells results in apoptosis and decreased viral reactivation, we assessed whether our three inhibitory methods would also reduce reactivation and virion production in HF cells. rKSHV.219-infected HF cells were either electroporated with IκBα-DN, treated with no inhibitor or treated with Bay11-7082 (Total, Post). We measured GFP forming units from supernatant collected at 24, 48, and 72 h post induction on 293 cells. Viral titer from induced HF cells increased to over 15,000 GFU by 72 h post-infection from basal levels (**Figure 3A**). With the addition of NFκB inhibitor Bay11-7082, both Total and Post, these titers dropped over three fold. Similarly low titers were seen from cells expressing IκBα-DN. These data demonstrate that NFκB inhibition can result in reduced viral titers from rKSHV.219-infected cells. To determine if these findings applied to other primary cells infected with HHV8, we performed the assay in MVEC cells and found similar results to HF cells (**Figure 3B**). Higher GFU were observed in MVEC, as these cells are a more permissive cell line to HHV8 infection and do have a low percentage of cells that undergo spontaneous lytic replication unlike HF as previously shown (Lagunoff et al., 2002).

Since inhibition of NFκB did not affect viral lytic reactivation but did result in decreased viral titers, we evaluated whether NFκB inhibition resulted in sequestration of virus in the cytoplasm of induced cells. We analyzed titers of cell-associated virus from induced rKSHV.219-infected HF cells following no treatment, treatment with Bay11-7082, or transfection with IκBα-DN. Cell-associated virus from Bay11-7082 treated cells and cells expressing IκBα-DN showed a similar drop in viral titer to virus collected from the supernatant, as compared to no treatment positive controls (**Figure 3C**). This suggests that the decrease in viral titer seen in cells treated with NFκB inhibitors was not secondary to sequestration of virus within the productively replicating cell but by potentially some other process.

**FIGURE 4:**
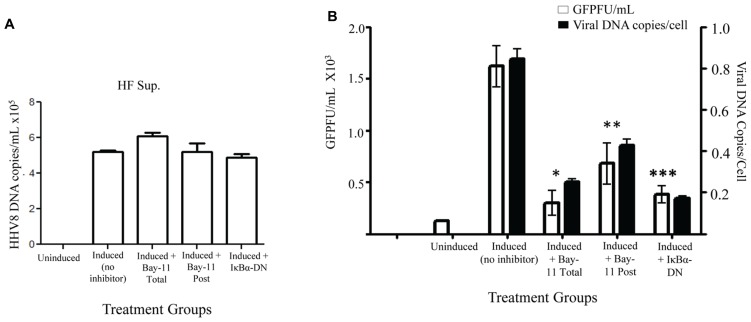
**Decreased viral entry by HHV8 virions in the presence of NFκB inhibitors**. rKSHV.219-infected HF cells were either mock induced (Uninduced) or induced to undergo lytic replication and then treated with Bay11-7082 prior to induction (Total), at the time of induction (Post), or transfected with IκBα-DN prior to induction. Supernatants were harvested 72 h post induction. **(A)** HHV8 DNA copy number was quantitated for cell supernatants by real time RT-PCR at 72 h post induction. There is no statistically significant difference between samples. Assay was done in triplicate and a representative of three experiments is shown. **(B)** DNA was extracted from supernatants from rKSHV.219 infected HF cells induced to undergo lytic replication, except for the Uninduced sample. When indicated, cells were either mock treated (No inhibitor, DMSO only), cotransfected with IκBα-DN or treated with Bay11-7082 prior to induction (Total) or at time of induction (Post). Viral DNA was quantitated by real time PCR (▪). Cell number was calculated by β-globin copy number. Supernatants were used in a viral titer assay on 293 cells to enumerate GFP forming units (□).Samples were assayed in triplicate and are representative of three experiments. For all experiments, 1 x 10^5^ cells were used. P values as follow for GFPF/mL as compared to induction only: *p < 0.043, **p < 0.0541, and ***p < 0.0349. For viral DNA copies/cell as compared to induction only: *p < 0.0489, **p < 0.0718, and ***p < 0.0340.

To further evaluate possible mechanisms for the observed reduction in viral titer caused by NFκB inhibition, we set out to measure HHV8 DNA copies in the cell supernatant. rKSHV.219-infected HF cells treated with vehicle control, Bay11-7082, or electroporated with IκBα-DN were induced and cell supernatant was harvested for DNA extraction. Prior to DNA extraction a known quantity of HSV gB was added to each sample, which was then treated with DNase; this step was to ensure all extracellular (non-virion associated) DNA was degraded. Quantitative real-time PCR was performed for HHV8 ORF73 and HSV gB. All samples were negative for gB DNA, suggesting adequate degradation of un-encapsulated DNA. We observed no difference between HHV8 virus particle-associated DNA from supernatants of induced rKSHV.219-infected HF cells and those treated with NFκB inhibitors, indicating that similar numbers of DNA containing virus particles were released from cells (**Figure 4A**). Thus far we have found that viral reactivation occurs in the presence of NFκB inhibitors and similar numbers of viral particles are produced, however, there is a significant drop in viral titers from both cell-associated and supernatant of cells induced to undergo productive replication in the presence of NFκB inhibitors.

In order to distinguish between defects in HHV8 viral particle binding and entry versus defects in subsequent steps in viral infection, we infected permissive 293 cells with supernatants from induced rKSHV.219-infected HF cells in the presence of Bay11-7082 or electroporated with IκBα-DN. We harvested virions from supernatant, as described above, and then infected 293 cells. Four hours after infection the cells were washed thrice with cold PBS and the last wash and cell pellet were subjected to quantitative PCR to enumerate HHV8 DNA found in the target 293 cells. The last wash in all groups did not amplify viral DNA. The number of viral DNA copies found per cell at four hours post-infection was dramatically lower in cells treated with Bay11-7082 or IκBα-DN, suggesting a block in virus binding or entry into target cells when virus is produced in the presence of an NFκB inhibitor (**Figure 4B**). Viral DNA copies per cell correlated with viral titer, quantitated by a viral titer assay performed with aliquots of the same supernatant on 293 cells. Taken together, these data suggest that NFκB inhibition in HF cells causes defects in HHV8 binding to target cells and/or viral entry, rather than defects in virion assembly.

### IDENTIFICATION OF HHV8 GENES AFFECTED BY NFκB INHIBITORS

**FIGURE 5:**
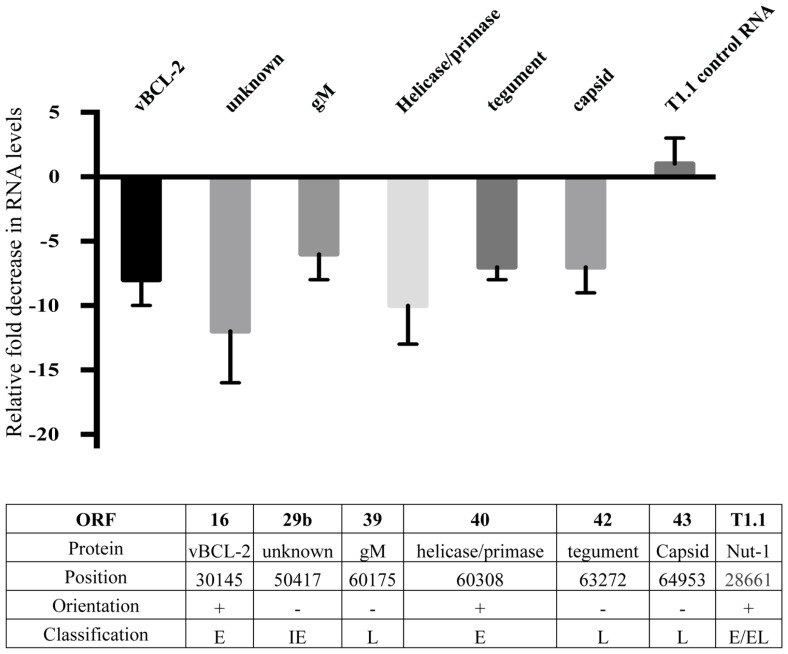
**Down regulation of HHV8 transcripts**. Real time RT-PCR analysis of RNA harvested from induced rKSHV.219 infected HF cells. Cells were either treated with DMSO alone (No Inhibitor) or Bay11-7082 and induced to undergo lytic replication. RNA was extracted and gene expression quantitated by real time RT-PCR. All values represent fold down regulation relative to No Inhibitor rKSHV.219 infected HF cells, which was set to 1. RNA levels were normalized using ribosomal RNA. Information below table identifies open reading frame, protein name, position in HHV8 genome, NFκB consensus site upstream of gene, orientation of gene, and immediate early (IE), early (E), or late (L) gene classification.

In order to further examine possible HHV8 genes that may be involved in defective binding or entry into target cells, we performed HHV8 genome wide RNA screening to identify qualitative differences in gene expression. We found a marked decrease in expression of several HHV8 lytic genes (data not shown). Notably, RNA levels of gM, ORF42, and ORF43 were substantially decreased in treated cells. We performed semi-quantitative real time PCR on RNA harvested from the Induced (no inhibitor) and Induced + Bay11-7082 treatment groups to confirm these qualitative results. Expression of all HHV8 RNA was not statistically significant between treatment and control groups for the majority of genes screened. However, when NFκB activity was inhibited, we observed transcriptional down regulation of ORF16, 29b, 39, 40, 42, and 43 (**Figure 5**). There was no statistically significant difference in T1.1 expression affected by NFκB inhibition, confirming previous results showing no difference in RFP expression in control versus Bay11-7082 groups when infected cells were induced to undergo productive replication (**Figures 2B,C**). The lower panel of **Figure 5** shows, for each gene, the encoded protein, genome position, orientation and classification. Because of its role in binding and entry, the gM protein was of particular interest given that we had found that viral particles produced in the presence of NFκB appeared to be inhibited at the binding and entry step. Additionally, despite the down regulation of viral helicase/primase there was no measurable difference of viral progeny production, which suggests that expression of these proteins may not be rate limiting in viral replication.

### RESTORATION OF INFECTIVITY BY COMPLEMENTATION WITH gM

**FIGURE 6:**
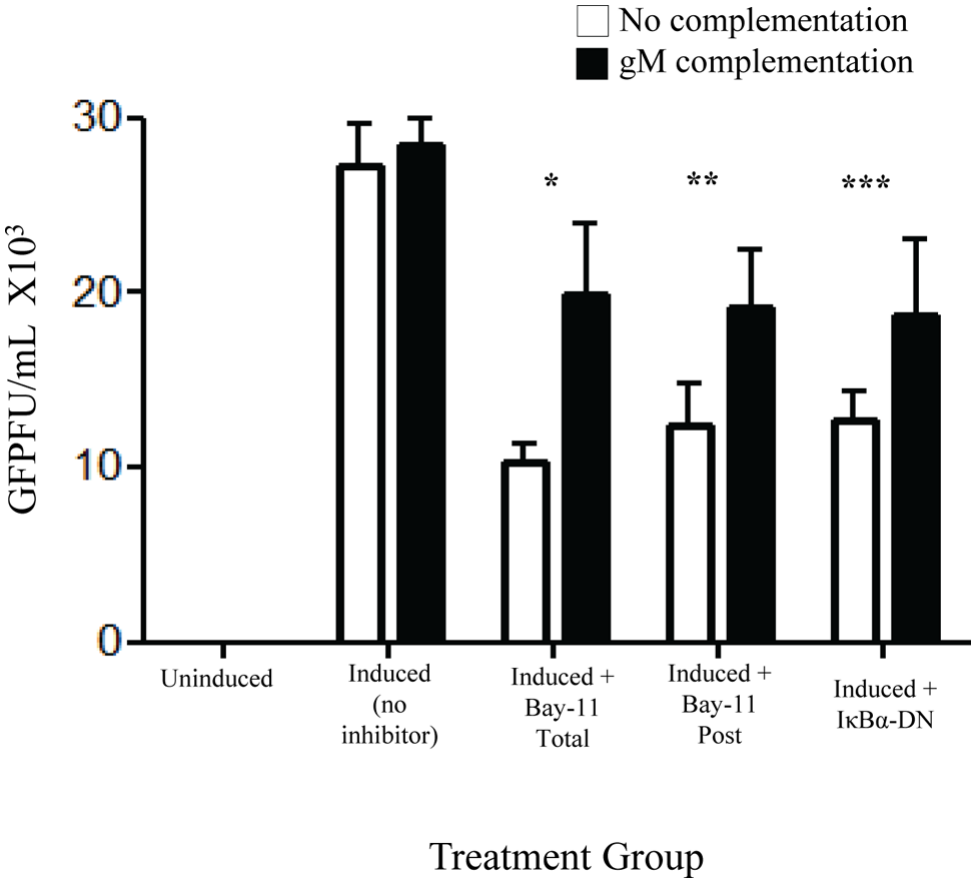
**gM complementation partially reverses loss of infectivity that results from NFκB inhibition**. rKSHV.219 infected HF cells were transfected with either vector alone (□) or pIRES-2-gM (▪) and either mock induced or induced to undergo lytic replication. They were then either mock treated (No inhibitor), transfected with IκBα-DN or treated with Bay11-7082 prior to induction (Total) or at time of induction (Post). Supernatants were harvested and GFP forming units on 293 were measured. Samples were run in triplicate and are representative of three separate experiments. Error bars represent standard deviations of the mean. *p < 0.0029; **p < 0.0092; ***p < 0.0202. 1 × 10^5^ cells were used per group in this assay.

Of the identified down regulated viral genes, we tested whether complementation with gM, a viral envelope protein involved in viral binding and entry (Koyano et al., 2003; May et al., 2005), could, at least in part, restore viral infectivity. An expression vector with an internal ribosome entry site, independent of NFκB, and including the gM open reading frame was transfected into rKSHV.219-infected HF cells. Cells were subsequently either mock treated, electroporated with IκBα-DN, or treated with Bay11-7082 24 h prior to or after induction. Following induction of viral replication we quantitated the relative amount of infectious virions produced by measuring GFP forming units on 293 cells. Introduction of gM into infected HF cells induced to undergo viral reactivation partially restored viral infectivity that is reduced by NFκB inhibitors (**Figure 6**). Thus, the block to viral binding and/or entry when NFκB is inhibited may be explained in part by insufficient levels of gM in progeny virus particles.

## DISCUSSION

These data indicate that the HHV8 lytic program in primary human foreskin fibroblasts and primary microvascular endothelial cells requires NFκB, a cellular transcription factor, to produce infection competent virions. Inhibition of NFκB during lytic replication of HHV8 in primary HF or MVEC leads to a dramatic decrease in infectious virions. Paradoxically, the decline in viral titer did not correlate with a decrease in viral particles released, as similar numbers of viral particles were enumerated between treatment groups. Virions produced in the presence of NFκB inhibitors showed a defect in binding and/or entry. This lack of infectivity correlated with reduced expression of the viral envelope protein gM (ORF39), as well as tegument (ORF42) and capsid (ORF43) proteins. These genes, which are in the negative orientation, have NFκB consensus sites upstream within ORF 44. Interestingly, we observed a decrease in RNA expression of vBCL-2, a proto-oncogene expressed by numerous gammaherpesvirus during latency in B cells (Sarid et al., 1997). This protein is important in the anti-apoptotic pathway of HHV8-related lymphoma, such as PEL, demonstrating cell-specific differences in NFκB activity effects on HHV8 gene expression.

Our data supports a model in which cells undergoing lytic replication require up-regulation of NFκB for effective expression of multiple viral proteins including gM, which is involved in virion production. Neither the inhibitor nor the DN completely shut down production of NFκB enhanced genes but they did lead to down-regulation at the transcriptional level, suggesting that these genes are most likely expressed despite the lack of NFκB. There may be a minimum protein concentration required for gM in order to generate infectious virions, which cannot be reached without full activation of NFκB. The down regulation of gM results in normal virion production but inefficient binding and/or entry into target cells. Our findings are consistent with those found by Grossmann and Ganem (2008) and Sadagopan et al. (2007) in which NFκB activity did rise with viral infection but was not involved in infection, establishment of latency, or viral reactivation. However, our findings demonstrate that down regulation of NFκB does result in defective viral particles.

Our results show that NFκB is required for production of infectious virions; however, NFκB levels did not influence entry into lytic replication. In a previous report, NFκB inhibitors initiated viral reactivation in PEL cells, although virion production and infectivity were not addressed (Sgarbanti et al., 2004). In our current study in HF cells, we observed no viral reactivation in the presence of NFκB inhibitors without the viral transactivator ORF50. Differences in these observations may be a result of the cell types used, as PEL cells are transformed cells that require NFκB for survival, whereas the primary cells we used do not, thus demonstrating a clear difference in cell biology ultimately influencing viral reactivation. These findings are in agreement with studies published by Grossmann and Ganem (2008) suggesting a true difference in cell biology between transformed and primary cells exists, as demonstrated by HF cells. However, human umbilical vein endothelial (HuVEC) cells do demonstrate an increase in lytic replication with inhibition of NFκB. As suggested by the authors this may be a reflection of cellular context as HuVECs support lytic replication at baseline without introduction of the viral transactivator, in contrast to HF cells which produce very low to undetectable levels of lytic reactivation (Lagunoff et al., 2002; Vieira and O’Hearn, 2004).

We have also shown that HHV8 viral particles produced in the presence of NFκB inhibitors are impaired in their ability to bind and/or enter target cells. The defect in binding and entry was partially corrected by the introduction of gM in trans to induced cells suggesting that this protein is a major player in viral infectivity. A previous report suggests that HHV8 particles produced in the absence of NFκB are able to enter target cells but unable to establish *de novo* infection (Keller et al., 2006). However, in that study the authors used an extremely high MOI on the order of 5,400, which makes comparisons difficult.

Viruses often utilize the NFκB pathway for viral gene expression and/or to promote survival of infected cells. EBV latent membrane protein-1 (LMP-1) induces abnormal NFκB activation and is associated with viral transformation of infected B cells (Luftig et al., 2004; Stewart et al., 2004). Herpes simplex virus (HSV) requires increased levels of NFκB activity in order to replicate and release virions. Blockade of NFκB activity during HSV replication leads to cellular apoptosis before virions can mature and exit the infected cell (Gregory et al., 2004). CMV immediate early gene expression can be initiated by NFκB leading to viral reactivation. In fact, signaling by TNF in patients following organ and bone marrow transplantation leads to potent activation of NFκB, which initiates immediate early gene expression and CMV reactivation resulting in profound morbidity and mortality (Hummel et al., 2001; Hummel and Abecassis, 2002). Our data add to this body of literature in which NFκB is utilized by the virus for its lifecycle.

Our findings suggest that using different strategies of NFκB inhibition may be a valid therapeutic strategy in patients with uncontrolled viremia, such as in patients receiving immunosuppressive therapy; AIDS patients; and at risk patients who have an increased likelihood of developing HHV8 associated diseases including PEL and Multicentric Castleman’s disease, both of which have high levels of lytic viral replication. Additionally, inhibitors of NFκB such as Bortezomib, a proteasome inhibitor associated with increased IκBα and β levels in treated cells, may be useful in decreasing the consequences of HHV8 replication (Matta and Chaudhary, 2005).

## Conflict of Interest Statement

The authors declare that the research was conducted in the absence of any commercial or financial relationships that could be construed as a potential conflict of interest.

## SUPPLEMENTARY MATERIAL

The Supplementary Material for this article can be found online at: http://www.frontiersin.org/journal/10.3389/fmicb.2014.00129/abstract
