# Supplementary material for: Nuclear factor kappa B is required for the production of infectious human herpesvirus 8 virions
Source: Front Microbiol. 2014 Apr 4;5:129. doi: 10.3389/fmicb.2014.00129 (PMC4006053; doi:10.3389/fmicb.2014.00129)
Supplement: Supplementary file 1 [file Data_Sheet_1.PDF]

Supplementary Table 1. Primers used in the study.

PRIMER	FORWARD	REVERSE
ORF16	AATGGACGAGGACGTTTTGC	CGTTTAATCCACAGGCCATGA
ORF29b	CTCCATCCCTAAGCGGGAC	TTGACCCGGTGACTACCTG
ORF39	TGAAAACAGCAGCATTTCCAA	TACTGACTCGGTGGAAACC
ORF40	CGGTATGGGTCGCCTAACT	GTCAGGATGTGCGGTTCAAA
ORF41	GGCATGTTTAGGGCTCGTTC	GAGGCATGTTTAGGGCTCGTT
ORF42	TATACCGTGCATGACGAAGGCC	TCGCGCCAGAAAGACACAT
ORF43	TGGATATGGTGTCCTGAGAATAGG	GCTGGCTCCCGTTGTTGA
T1.1	CGCTTTTGGCAATATACCCATC	CAGGCCAATGTGGGAAAAGT
K8.1	CCGCGGTTCAGTCATCAAC	CCAATAACAGGCGACGAAGAG
